# Association of complete blood cell counts with metabolic syndrome in an elderly population

**DOI:** 10.1186/s12877-016-0182-9

**Published:** 2016-01-13

**Authors:** Peng-Fei Li, Jin-Shuen Chen, Jin-Biou Chang, Hsiao-Wen Chang, Chung-Ze Wu, Tsung-Ju Chuang, Chia-Luen Huang, Dee Pei, Chang-Hsun Hsieh, Yen-Lin Chen

**Affiliations:** Division of Endocrinology and Metabolism, Department of Internal Medicine, Tri-Service General Hospital, National Defense Medical Center, Taipei, Taiwan; Division of Nephrology, Department of Internal Medicine, Tri-Service General Hospital, National Defense Medical Center, Taipei, Taiwan, R.O.C.; Department of Pathology, Division of Clinical Pathology, Tri-Service General Hospital, National Defense Medical Center, Taipei, Taiwan, R.O.C.; Division of Endocrinology, Department of Internal Medicine, Shuang-Ho Hospital, School of Medicine, College of Medicine, Taipei Medical University, Taipei, Taiwan, R.O.C.; Department of Internal Medicine, Cardinal Tien Hospital, School of Medicine, Fu-Jen Catholic University, New Taipei City, Taiwan; Department of Pathology, Cardinal Tien Hospital, School of Medicine, Fu-Jen Catholic University, New Taipei City, Taiwan

**Keywords:** Metabolic syndrome components, Hematogram parameters, Elderly population

## Abstract

**Background:**

Metabolic syndrome’s (MetS) role in predicting cardiovascular diseases and diabetes has been confirmed in many large cohort studies. Nontraditionally, hematogram components are significantly related to MetS in many different age groups. However, little is known about its role among the elderly.

**Methods:**

We enrolled 18,907 subjects over the age of 65 years who underwent regular health examinations. They were divided into three groups according to age: young old (YO: ≥ 65 and < 74 years old), old old (OO: ≥ 75 and < 84 years old), and oldest old (ODO: ≥ 85 years old). The MetS components were determined, and correlations between MetS and hematogram components were evaluated using Pearson and multivariate linear regression analyses. The hematogram components were the independent variables evaluated separately against the dependent variable (MetS components).

**Results:**

While SBP and HDL-C increased, most other MetS and hematogram parameters decreased in men with age. Fewer significant differences were noted among the women. In the YO and OO groups for both genders, the subjects with MetS had higher WBC and Hb. None of the hematogram components were different for subjects with or without MetS in the ODO group. Multiple regression results show that most of the relationships between hematogram and MetS components disappeared in the ODO groups. The WBC levels were mainly correlated with WC and TG. At the same time, Hb was associated with BP, FPG, and LDL-C. Compared to WBC and Hb, PLT was least related to MetS, except in the cases of LDL-C and TG. Among the MetS components, BMI, LDL-C, and TG were consistently related to all the hematogram components in YO and OO men. However, only TG had the same consistency among YO and OO women.

**Conclusions:**

This study’s three major findings are as follows: WBC and Hb are associated with MetS, even among the YO and OO groups, regardless of gender; among the three hematogram components, Hb had the strongest and PLT had the weakest correlation with MetS; and TG is not the only component with relatively higher *r* values, and it is related to all hematogram components.

## Background

Cardiovascular diseases (CVD) and diabetes have always been among the top five causes of death in Taiwan, as well as in many other countries. They cause tremendous burden, not only for the affected individuals but also for society as a whole. Since the prevalence of obesity has simultaneously increased, it is considered to be a main cause of this phenomenon [1].

Early in 1964, the clustering of hyperglycemia, hyperlipidemia, hypertension, and central obesity was found to be related to the aforementioned two diseases. In 1999, to perform early detection of the subjects most at risk for these diseases, the World Health Organization (WHO) proposed the term “metabolic syndrome (MetS)” to denote this clustering. Subsequently, many large-scale studies were performed and repeatedly confirmed the predictive power of MetS. At the same time, several other abnormalities, such as microalbuminuria, inflammation markers, and adipocytokines were found to have strong correlations with CVD and diabetes [2, 3]. Contrary to the MetS components, these markers are considered to be “non-traditional components” [4–8]. For example, white blood cell (WBC) count was first found to be associated with MetS by Nagasawa et al. This is not completely surprising, since WBC itself is a marker of inflammation [9]. What is more interesting is that hemoglobin (Hb), platelet (PLT) count, and mean platelet volume (MPV) all had similar relationships. This evidence strongly implies an association between hematogram components and chronic inflammation [10–14]. Although a number of studies have been published with younger adults [4–12], little is known about the similar relationships between hematogram and MetS components among the elderly.

Due to the National Health Insurance Policy, modern Taiwanese have a longer life expectancy than ever before. At present, 14 % of the population is over 65 years old, which marks this as an “aging society”. Additionally, the increased incidence of MetS among the elderly will inevitably lead to more CVD and T2D. This is a major issue that will have to be dealt with by health providers and policy makers alike. Since a hematogram is a readily available, inexpensive test that can be performed even in basic medical units, it would be a very good tool for detecting subjects who are at high risk of developing MetS.

It is generally agreed that health problems may vary according to age group, and this is especially true among the elderly. Due to these different relationships between risk factors and outcomes, previous studies have further classified the elderly into three groups: young old (YO: ≥ 65 and < 75), old old (OO: ≥ 75 and < 85), and oldest old (ODO ≥ 85) [15, 16]. In this cross-sectional study, our purpose was first to demonstrate the demographic information of the hematogram parameters and further analyze their relationships with MetS components among the three elderly groups. It is expected that the present study’s results will enable a greater understanding of the roles of MetS hematogram components.

## Method

### Study population

MJ Health Screening Centers are privately owned clinics located throughout Taiwan that only provide regular health examinations to their members. The participants in this study were enrolled when they underwent their routine health checkups at this clinic. Anonymity was ensured for, and informed consent was obtained from, all study participants. The study protocol was approved by the institutional review board of the MJ Health Screening Center, and the data obtained were used for research purposes only. We randomly selected 36,169 subjects who were over 65 years old during the sampling period, from 1999 to 2008. We excluded 3347 subjects who visited only once during the sampling period. We used more stringent exclusion criteria to make our results more reliable. We excluded subjects with past histories of hypertension, T2D, hyperlipidemia, cardiovascular events, renal function impairment (plasma creatinine >1.2 mg/dl), established bone marrow or hematology diseases, established malignancy or gastrointestinal tract bleeding, and recent infectious disease. Those who were taking medications (such as antibiotics, immune suppression agents, steroids, hormonal treatment, etc.) known to affect complete blood cell counts or MetS components were also excluded (*n* = 11,562). Data from 2353 more subjects who had missing values for MetS components, hematogram components, and other related data were removed from the analysis, leaving a total of 18,907 eligible subjects. To further study the different elderly groups, we grouped subjects according to their ages as YO, OO, and ODO groups [17].

### Anthropometric measurements and general data

A standard checkup protocol was followed at the MJ clinic. The senior nursing staff used a questionnaire to obtain the subject’s medical history, including information on any current medications. Then, complete physical examinations were performed. Waist circumference (WC) was measured horizontally at the level of the natural waist, which was identified as the level at the hollow molding of the laterally concave trunk. Body mass index (BMI) was calculated as a subject’s body weight (kg) divided by the square of the subject’s height (m). Both systolic blood pressure (SBP) and diastolic blood pressure (DBP) were measured by the nursing staff using a standard mercury sphygmomanometer that was fitted on the right arm of each seated subject. Laboratory measurements, including blood sample analysis, were conducted after the subjects fasted for 10 h. The blood samples were drawn from the antecubital vein for biochemical analysis. Plasma was separated from the blood within 1 h, it was stored at −30 °C, and then it was analyzed for both FPG and lipid profiles. FPG was detected using a glucose oxidase method (YSI 203 glucose analyzer, Scientific Division, Yellow Springs Instruments, Yellow Springs, OH). Total cholesterol and triglycerides (TG) were measured using the dry, multilayer analytical slide method in the Fuji Dri-Chem 3000 analyzer (Fuji Photo Film, Minato-Ku, Tokyo, Japan). Serum high-density lipoprotein cholesterol (HDL-C) and low-density lipoprotein cholesterol (LDL-C) concentrations were analyzed using an enzymatic cholesterol assay following dextran sulfate precipitation. WBC, Hb, and PLT were measured with an Abbott Cell Dyn 3000 hematology analyzer (Abbott Laboratories, Abbott Park, IL, USA).

### Definition of metabolic syndrome

We used a slightly modified version of the 2009 harmonized MetS criteria [18]. WC was ≥ 90 and 80 cm in Taiwanese men and women, respectively [19]. The other four criteria were the same; namely, SBP ≥130 mmHg or DBP ≥85 mmHg, TG ≥150 mg/dL, FPG ≥100 mg/dL, and HDL ≤ 40 and 50 mg/dL for men and women. Subjects had to meet at least three criteria to be diagnosed as MetS.

### Statistical analysis

The data in this study are presented as mean ± standard deviation. All data were tested for normal distribution with the Kolmogorov–Smirnov test, and the homogeneity of their variances was assessed with Levene’s test. A *t*–test was used to evaluate the differences between two groups. When comparing the differences between three groups, a one-way ANOVA was used. For post hoc comparisons, the Bonferroni test was applied. Correlations between MetS components and hematogram were evaluated using the Pearson correlation. Multivariate linear regression analysis was performed to confirm if hematogram was independently related to MetS components. A *p*-value (two-sided) < 0.05 was considered to be significant. All statistical analyses were performed using SPSS 18.0 software (SPSS Inc., Chicago, IL).

## Results

There were 18,907 subjects enrolled in this study. Table 1 shows the demographic data for the different groups and allows an examination of the effects of aging on the observational parameters. In general, BMI, DBP, TC, LDL-C, TG, Hb, and PLT became significantly lower, and SBP and HDL-C became significantly higher in men as they aged. As for women, fewer significant differences were noted.

**Table 1:** Demographic data for the different elderly groups

	Young-old	Old-old	Oldest-old	*P* value
Male				
n	7648	1963	121	
Age (years)	68.7 ± 2.8	77.8 ± 2.5	87.0 ± 2.2	<0.001
WC (cm)	85.1 ± 9.1	84.9 ± 9.6	84.6 ± 10.1	0.492
BMI (kg/m^2^)	23.8 ± 3.1	23.2 ± 3.2	22.8 ± 3.2	<0.001
SBP (mmHg)	135.6 ± 20.3	139.3 ± 20.2	139.5 ± 22.4	<0.001
DBP (mmHg)	77.3 ± 11.7	75.2 ± 11.9	73.4 ± 12.9	<0.001
FPG (mg/dl)	109.8 ± 32.5	110.4 ± 32.1	105.5 ± 17.9	0.238
TC (mg/dl)	199.8 ± 36.4	194.3 ± 35.4	190.8 ± 35.0	<0.001
HDL-C (mg/dl)	49.3 ± 13.9	50.4 ± 14.7	51.7 ± 16.0	0.003
LDL-C (mg/dl)	125.0 ± 32.7	119.3 ± 31.3	116.9 ± 30.2	<0.001
TG (mg/dl)	127.7 ± 68.1	123.1 ± 65.3	110.9 ± 50.6	0.001
LogTg	2.1 ± 0.2	2.0 ± 0.2	2.0 ± 0.2	<0.001
WBC (x10^3^/uL)	6.5 ± 1.8	6.5 ± 1.9	6.7 ± 1.8	0.461
Hemoglobin (g/dl)	14.7 ± 1.3	14.3 ± 1.4	13.8 ± 1.6	<0.001
Platelet (x10^3^/uL)	213.1 ± 55.7	202.9 ± 57.0	197.6 ± 52.5	<0.001
Female				
n	7521	1573	81	
Age (years)	68.5 ± 2.7	77.8 ± 2.6	86.8 ± 2.0	<0.001
WC (cm)	80.1 ± 8.9	82.1 ± 9.6	83.5 ± 9.1	<0.001
BMI (kg/m^2^)	24.5 ± 3.6	24.0 ± 3.7	23.5 ± 4.0	<0.001
SBP (mmHg)	139.9 ± 20.8	145.9 ± 21.2	149.5 ± 19.4	<0.001
DBP (mmHg)	76.8 ± 11.8	76.2 ± 12.3	76.8 ± 11.5	0.230
FPG (mg/dl)	110.0 ± 33.5	110.1 ± 29.6	109.4 ± 26.7	0.976
TC (mg/dl)	214.3 ± 38.5	210.5 ± 36.9	212.7 ± 42.4	0.002
HDL-C (mg/dl)	57.0 ± 15.1	57.0 ± 15.8	56.8 ± 16.6	0.976
LDL-C (mg/dl)	129.6 ± 34.5	125.8 ± 32.5	127.3 ± 37.6	<0.001
TG (mg/dl)	138.2 ± 69.2	139.5 ± 68.5	143.0 ± 65.9	0.668
LogTg	2.1 ± 0.2	2.1 ± 0.2	2.1 ± 0.2	0.381
WBC (x10^3^/uL)	6.2 ± 1.7	6.3 ± 1.8	6.7 ± 2.0	0.002
Hemoglobin (g/dl)	13.3 ± 1.1	13.0 ± 1.3	12.9 ± 1.3	<0.001
Platelet (x10^3^/uL)	231.2 ± 56.5	226.2 ± 59.4	213.1 ± 59.4	<0.001

*BMI* body mass index, *WC* waist circumference, *SBP* systolic blood pressure, *DBP* diastolic blood pressure, *FPG* fasting plasma glucose, *TC* total cholesterol, *HDL-C* high-density lipoprotein cholesterol, *LDL-C* low-density lipoprotein cholesterol, *TG* triglyceride, *LogTG* log transformed triglyceride, *WBC* white blood cell count

Data are shown as mean ± SD

Hb and PLT were lower, while WC, SBP, and WBC were higher for the ODO group of women.

Table 2 shows the differences of hematogram components in subjects with and without MetS. In the YO and OO groups for both sexes, not surprisingly the subjects who had MetS had higher WBC and Hb. PLT levels were not higher for the subjects with MetS, except for the YO group in females. Interestingly, none of the hematogram components were different for the subjects with and without MetS in the ODO group.

**Table 2:** Differences in hematogram components between subjects with or without metabolic syndrome

	Young-old			Old-old			Oldest-old		
	MetS(−)	MetS(+)	*p* value	MetS(−)	MetS(+)	*p* value	MetS(−)	MetS(+)	*p* value
Male									
n	5049	2599		1273	690		83	38	
WBC	6.3 ± 1.7	6.8 ± 1.8	<0.001	6.2 ± 1.8	6.9 ± 2.0	<0.001	6.6 ± 2.0	6.9 ± 1.5	0.393
Hb	14.6 ± 1.3	15.0 ± 1.3	<0.001	14.1 ± 1.4	14.5 ± 1.5	<0.001	13.6 ± 1.6	14.1 ± 1.5	0.105
PLT	212.5 ± 55.8	214.3 ± 55.5	0.168	201.4 ± 58.7	205.7 ± 53.7	0.111	198.0 ± 53.1	196.9 ± 51.7	0.914
Female									
n	4004	3517		718	855		30	51	
WBC	5.9 ± 1.6	6.6 ± 1.8	<0.001	5.9 ± 1.7	6.6 ± 1.9	<0.001	6.3 ± 2.0	6.9 ± 2.0	0.203
Hb	13.2 ± 1.1	13.4 ± 1.2	<0.001	12.8 ± 1.2	13.2 ± 1.3	<0.001	12.6 ± 1.3	13.0 ± 1.2	0.107
PLT	226.3 ± 55.3	236.8 ± 57.4	<0.001	223.0 ± 58.9	228.9 ± 59.6	0.050	202.8 ± 53.0	219.2 ± 62.5	0.233

*MetS(−)* without metabolic syndrome, *MetS(+)* with metabolic syndrome, *WBC* white blood cell count, *Hb* hemoglobin, *PLT* platelet count

Data are shown as mean ± SD

The results of the Pearson’s correlation and multiple regression are shown in Tables 3 and 4, respectively. In multiple regression, the hematogram components (WBC, Hb, and PLT) were taken as independent variables and were evaluated separately against the dependent variable (MetS components). Using this method, the effects from the individual hematogram components could be adjusted. In general, most of the relationships between hematogram and MetS disappeared in the ODO groups. The WBC levels were mainly correlated with WC and TG. At the same time, Hb was found to be associated with BP, FPG, and LDL-C. Compared to WBC and Hb, PLT was least related to MetS, except in the cases of LDL-C and TG. Among the MetS components, it is interesting to note that BMI, LDL-C, and TG were consistently related to all hematogram components for YO and OO men. However, only TG had the same consistency for YO and OO women.

**Table 3:** Univariate analysis of hematogram and metabolic syndrome components

	Young-old			Old-old			Oldest-old		
	WBC	Hb	PLT	WBC	Hb	PLT	WBC	Hb	PLT
Male									
Age	NS	−0.091	−0.062	NS	−0.108	NS	NS	NS	NS
BMI	0.096	0.198	−0.044	0.060	0.221	−0.097	NS	0.259	NS
WC	0.152	0.195	NS	0.113	0.215	NS	NS	0.219	NS
SBP	0.079	0.084	NS	0.049	0.090	NS	NS	0.263	NS
DBP	0.049	0.170	NS	NS	0.195	NS	NS	0.396	NS
FPG	0.072	0.064	0.025	NS	0.062	NS	NS	NS	NS
HDL-C	−0.129	NS	NS	−0.171	−0.063	−0.051	−0.218	NS	NS
LDL-C	0.084	0.169	0.091	0.078	0.212	0.084	NS	0.240	NS
TG	0.185	0.158	0.109	0.218	0.175	0.118	NS	0.202	NS
Female									
Age	0.040	−0.068	−0.038	NS	NS	NS	NS	−0.267	NS
BMI	0.173	0.130	NS	0.151	0.156	NS	NS	0.226	NS
WC	0.205	0.099	0.039	0.193	0.120	NS	NS	0.257	NS
SBP	0.104	0.062	NS	0.073	0.062	NS	NS	NS	NS
DBP	0.070	0.157	NS	0.083	0.192	NS	NS	NS	−0.325
FPG	0.122	0.122	0.034	0.111	0.085	0.056	NS	NS	NS
HDL-C	−0.113	0.023	−0.026	−0.126	NS	NS	−0.308	NS	NS
LDL-C	0.046	0.131	0.117	0.054	0.146	0.057	NS	0.221	NS
TG	0.213	0.121	0.142	0.179	0.125	0.112	0.274	NS	NS

*BMI* body mass index, *WC* waist circumference, *SBP* systolic blood pressure, *DBP*diastolic blood pressure, *FPG* fasting plasma glucose, *TC* total cholesterol, *HDL-C* high-density lipoprotein cholesterol, *LDL-C* low-density lipoprotein cholesterol, *TG* triglyceride, *NS* non-significant

Data are shown as the *r* value when *p* value < 0.05

**Table 4:** Multivariate analysis of hematogram and metabolic syndrome components

	Young-old			Old-old			Oldest-old		
	WBC	Hb	PLT	WBC	Hb	PLT	WBC	Hb	PLT
Male									
Age	---	---	−0.060	---	−0.077	---	---	---	---
BMI	0.086	0.092	−0.091	0.091	0.131	−0.166	---	NS	---
WC	0.223	0.058	---	0.172	NS	---	---	NS	---
SBP	0.074	−0.112	---	NS	−0.085	---	---	NS	---
DBP	NS	0.206	---	---	0.208	---	---	0.398	---
FPG	0.029	0.037	NS	---	NS	---	---	---	---
HDL-C	−0.053	---	---	−0.103	0.066	−0.052	−0.218	---	---
LDL-C	0.074	0.148	0.090	0.057	0.169	0.083	---	NS	---
TG	0.137	0.093	0.127	0.168	0.111	0.140	---	NS	---
Female									
Age	NS	−0.050	−0.039	---	---	---	---	NS	---
BMI	NS	0.119	---	NS	0.125	---	---	NS	---
WC	0.138	NS	NS	0.165	NS	---	---	NS	---
SBP	0.071	−0.115	---	NS	−0.119	---	---	---	---
DBP	NS	0.201	---	NS	0.240	---	---	---	−0.325
FPG	0.066	0.104	NS	0.067	0.053	NS	---	---	---
HDL-C	NS	0.109	0.040	NS	---	---	NS	---	---
LDL-C	0.032	0.108	0.110	NS	0.126	0.051	-	NS	---
TG	0.162	0.119	0.153	0.127	0.090	0.107	NS	---	---

--- indicates non-inclusion in the regression model, *BMI* body mass index, *WC* waist circumference, *SBP* systolic blood pressure, *DBP* diastolic blood pressure, *FPG* fasting plasma glucose, *TC* total cholesterol, *HDL-C* high-density lipoprotein cholesterol, *LDL-C* low-density lipoprotein cholesterol, *TG* triglyceride, *NS* non-significant

Data are shown as the β value when *p* value < 0.05

## Discussion

To our knowledge, the present study is the first to focus on the relationships between hematogram and MetS components among these three elderly groups. WBC and Hb were correlated with most of the MetS components in men, except for the ODO group. Fewer significant relationships were found among the women, especially those in the OO group. Our data have also shown that, compared to WBC and PLT, Hb was more strongly related to MetS. Finally, among the MetS factors, WC had the highest *r* value in the multiple regression with WBC in males. At the same time, DBP was the most significant component to be related to Hb, with an average *r* value of 0.2.

Because of the complexity of the results, we will discuss the significant findings between MetS and hematogram separately in the following sections so they may be expressed more clearly.

1. The relationship between adiposity and hematogram:
  In the present study, we found interesting relationships between BMI, WC, and hematogram. In short, BMI had a negative correlation with hematogram, except in the ODO group. On the contrary, WC was positively correlated with it. Since obesity increases both the size and numbers of adipocytes resulting in increased macrophage infiltration and pro-inflammation status, theoretically the correlations between adiposity and hematogram should be positive [14]. This discrepancy in our study could be readily explained by the fact that lean body mass decreases as the subject gets older. At the same time, abdominal fat increases. Thus, BMI decreases with age, but WC changes in a contrasting direction.
2. The relationship between WBC and MetS components:
  In this study, we found positive correlations between WBC and MetS components in the YO and OO, but not the ODO, groups. Again, these relationships are expected to be due to the effects of proinflammatory cytokines (such as tumor necrosis factor-alpha and interleukin-6), which are secreted by activated macrophages. The macrophage itself is one type of WBC that has been shown to be significantly synthesized in adipose tissue [14]. Several cross-sectional studies have also confirmed our results [20–22]. For example, Tao et al. reported that WBC counts were positively associated with MetS among the young adult (between 20–50 years old) population of Beijing [23]. Generally speaking, this finding is in line with the findings from other major studies. However, no similar report exists for the three elder groups.
3. The relationship between Hb and MetS components:
  Hb was significantly related to WBC and MetS components. These relationships have been reported with many different groups [24, 25]. Again, most of them were only limited to adults, rather than specifically the elderly. One of the most important longitudinal studies was done by Laudisio et al., who showed that MetS was associated with higher Hb levels in a 6-year follow-up period in an older population (≧65 years old) [26]. However, their study placed no particular emphasis on the three different elderly age groups. The role of insulin in stimulating erythropoiesis could be responsible for this relationship. Since insulin resistance is the core of MetS [27], it is not difficult to understand that subjects with MetS would have higher plasma insulin levels to stimulate all stages of erythropoiesis [28].
4. The relationship between PLT and MetS components:
  Among the three hematogram components, PLT had the weakest relationships with MetS, except in the cases of LDL-C and TG. PLT is known to play an important role in inflammation due to its effect of inducing the expression of cyclooxygenase-2 and prostanoids, which accelerate atherothrombosis and cause other features noted in MetS [29–31]. For example, Jesri et al. found that subjects with MetS have higher PLT counts after adjusting for variables such as age and gender [32]. At the same time, Kotani et al. also demonstrated that PLT counts may be biomarkers for MetS among Japanese women [33]. However, in the present study, no correlation between PLT and MetS was found, and this was inconsistent with the aforementioned pathophysiologies. One possible explanation for this discrepancy is that PLT is seriously influenced by age. Biino et al. showed that PLT reduces by 35 % in men and 25 % in women among the elderly [34]. Hence, the positive correlations between higher PLT and MetS may be masked by a physiological decline in PLT with age.
5. The uniqueness of TG among the other MetS components:
  It is interesting to note that among the five MetS components, TG was the only one correlated with all hematogram components. Since TG itself is a risk factor for CVD and T2D, this result is not surprising [35]. This positive finding is also consistent with the findings of other studies. Huang et al. showed that serum TG level was positively correlated with WBC counts in the group of Taipei residents who attended a regular hospital health check program [36]. Cohn et al. also reported that hypertriglyceridemia was strongly related to higher BW and lower levels of insulin sensitivity [37]. The possible mechanism for this relationship could be explained by the work of Feingold et al., who reported that TNF-α and IL-6 stimulate lipolysis and increase the flow of free fatty acids to the liver [38]. These increasing free fatty acids induce hepatic TG synthesis, thus causing hypertriglyceridemia. Contrary to the findings of our study, Purnamasari et al. found that the most important metabolic component was central obesity (56.7 %), followed by hypertension, hypertriglyceridemia, and hyperglycemia [39]. Our study showed that instead of central obesity, hypertriglyceridemia may be more important for MetS development among ODO groups.
6. The disappearance of most of the relationships in the ODO group:
  There is an important and intriguing finding in the present study. In the ODO group, all relationships between MetS and hematogram components, except for TG and LDL, disappeared. Since there are no apparent differences in either the MetS or hematogram components among this group compared to the two younger groups, the effects of aging on these components should not be responsible for this disappearance. The most likely explanation is that the ODO sample was quite small compared to those of the YO and OO groups. At the same time, all other significant *r* values were relatively weak. Therefore, the loss of significance in the ODO group could be rationally explained by combining these two factors.

However, our study is not without its limitations. First, all subjects were enrolled when they received their regular health examinations. Thus, the study members may all have been from a higher socio-economic background and consequently might have had lower risk of chronic inflammation diseases than that of the general population. However, the relationships we examined here should not be affected by socio-economic status.

Second, the ODO group was smaller compared to the other two groups, but this also reflects the distribution of the aged subjects in the real world. Third, the core of MetS is insulin resistance, which we were unable to measure due to the lack of plasma insulin levels in our present study. Finally, this is a retrospective study, and further prospective investigations are needed to support the present findings.

## Conclusion

In conclusion, this study found the following: 1. WBC and Hb were indeed associated with MetS, even in the YO and OO groups, regardless of gender; 2. Among the three hematogram components, Hb had the strongest and PLT the weakest correlation with MetS; and 3. TG is not the only component that had relatively high *r* values, and it is related to all the hematogram components.

## Abbreviations

MetS: metabolic syndrome
YO: young old
OO: old old
ODO: oldest old
WBC: white blood cell
Hb: hemoglobin
PLT: platelet
BMI: body mass index
SBP: systolic blood pressure
DBP: diastolic blood pressure
TG: triglycerides
HDL: high-density lipoprotein cholesterol
LDL: low-density lipoprotein cholesterol
